# Advances in Jellyfish Sting Mechanisms and Treatment Strategies

**DOI:** 10.3390/md23060231

**Published:** 2025-05-28

**Authors:** Bingbing Li, Yueyue Li, Zhiwen Qiu, Chuantao Zhang, Yue Li, Wei Li, Jishun Yang

**Affiliations:** 1 Naval Medical Center of PLA, Naval Medical University, Shanghai 200052, China; m18131452435@163.com (B.L.); yueyueli921@163.com (Y.L.); 2 Shanghai Key Laboratory of Nautical Medicine and Translation of Drugs and Medical Devices, Shanghai 200433, China; qzw0531@126.com; 3 School of Pharmacy, Bengbu Medical University, Bengbu 233000, China; zct13253@163.com; 4 School of Health Science and Engineering, University of Shanghai for Science and Technology, Shanghai 200093, China; 18337018029@163.com

**Keywords:** jellyfish stings, emission mechanism, biological activity, treatment

## Abstract

Jellyfish stings, as one of the most prevalent forms of marine injury, have increasingly become a subject of concern. Despite their simple morphology and structure, jellyfish possess a complex venom composition that can inflict varying degrees of damage on multiple human physiological systems. Consequently, the clinical symptoms associated with jellyfish stings are highly intricate. Although antivenoms have been developed for certain jellyfish species (e.g., *C. fleckeri*), specific antivenoms targeting the mechanisms of most jellyfish venoms remain understudied. To effectively prevent, treat, and cure jellyfish stings, we adhere to the principle of knowing their nature and their reasons. It is essential to investigate the emission mechanism of jellyfish nematocysts and the composition of their venom. Understanding these factors is crucial for the development of targeted treatment strategies. This review delves into the venom emission mechanism of jellyfish stinging cells, the symptoms resulting from jellyfish stings, and the comprehensive treatment strategies post-sting. It offers a scientific reference for comprehending jellyfish stings and establishes a theoretical foundation for subsequent research endeavors.

## 1. Introduction

Jellyfish, a primitive invertebrate zooplankton inhabiting aquatic ecosystems, encompass a diverse array of species and morphologies, commonly found across numerous marine regions. Nevertheless, the synergistic effects of global warming and the intensification of human fishing efforts have triggered substantial alterations in marine biodiversity, culminating in a concerning surge in jellyfish populations.

This phenomenon poses severe threats not only to coastal economic vitality but also to human activities and the integrity of marine ecosystems. The occurrence of jellyfish outbreaks leads to a significant surge in the population of jellyfish within the affected area, consequently elevating the likelihood of sting incidents. Globally, it is estimated that there are upwards of 150 million jellyfish stings each year [1]. Coastal nations such as Japan, Australia, Thailand, and Malaysia regularly report instances of jellyfish stings, with some cases proving fatal. From 2017 to 2019 alone, the seaside baths in Qinhuangdao City, Hebei Province, recorded a total of 2553 jellyfish stings [2].

As one of the high-risk groups susceptible to jellyfish stings, naval personnel are frequently the targets of such incidents. However, a significant knowledge gap exists among these individuals regarding the nature of jellyfish stings and the appropriate emergency response, which has become a matter of public concern. Urgent research on the mechanism and treatment of jellyfish sting poisoning is necessary, as the efficacy of its treatment remains unclear. In light of this, we initially elucidated the structure and emission mechanism of nematocyst cells in jellyfish to enhance our comprehension of their biological function and establish a foundation for the development of novel drugs, therapeutic approaches, and bionics. Furthermore, we have compiled a comprehensive summary of the composition of jellyfish venoms, the symptoms that arise from stings, and the various treatment modalities available, with the aim of providing scientific insights for future research on the prevention and management of jellyfish stings.

This paper reveals the underlying stimulation mechanisms and lethal effects of jellyfish stings, as well as the medications commonly used in their treatment. It offers theoretical guidance for the advancement of drug development targeting jellyfish envenomation and for the establishment of emergency management protocols for patients exhibiting severe reactions to stings.

## 2. Cnidocytes in Jellyfish: Unveiling the Mechanism of Stinging Emission

Jellyfish cnidocytes are fundamental to their biological adaptation, serving essential roles in predation, defense, and even modulating their locomotion speed. The sophisticated structure and effective operation of cnidocytes allow jellyfish to carve out a substantial ecological role in marine environments. As a result, understanding cnidocytes enhances our knowledge of the distinct biological features of jellyfish and lays the groundwork for investigating related coelenterates. Ongoing advancements in science and technology suggest that we may yet discover more functions of cnidocytes, which could provide fresh perspectives and deepen our insights into biological and ecological studies.

**Figure 1:**
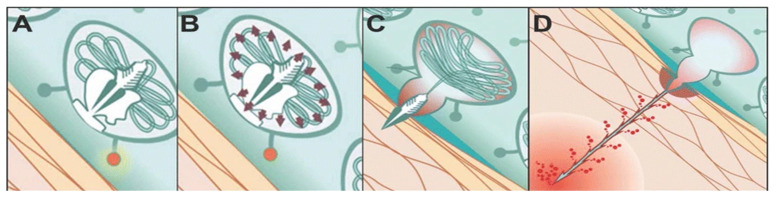
Schematic diagram of the cnidocyte emission process [5]. (**A**) The cnidocyst in a resting state. The cnidocyst’s cnidae are coiled and not discharged, remaining in a ready-to-fire state; (**B**) After being stimulated, the osmotic pressure inside the cnidocyst changes, and the pressure inside the cnidocyst rises. The cnidae start to prepare for eversion and discharge; (**C**) The cnidae start to evert and discharge. The top of the cnidocyst opens, and the cnidae are rapidly ejected outward; (**D**) The cnidae are completely discharged and penetrate target objects, and venom may be released simultaneously.

Jellyfish are adorned with a complex array of unique stinging cells that cluster around their tentacles and mouth regions. These cells, known as cnidocytes, are composed of nematocysts, needle-like structures, nuclei, and apical caps. Among them, nematocysts are elaborate organelles derived from the Golgi [3], featuring venomous threads (filaments) enclosed within pressurized capsules. The pressure capsule is composed of multiple proteins that form cysteine-rich microcollagens with a crosslinked wall created by intermolecular disulfide bonds, resulting in a three-dimensional polymer capable of withstanding exceedingly high internal pressures (1.5 × 10^7^ Pa). When the nematocyst is stimulated (when the pressure inside the nematocyst exceeds 1.5 × 10^7^ Pa), it is expelled from the sac and launched with an acceleration of 5.41 × 10^6^ g. The deployed nematocyst can extend to a length 100 times the diameter of the sac [4], allowing for venom release through the nematocyst (Figure 1A–D) [5]. The extension driving force is determined by both concentration-dependent pressure and annular cross-sectional area at the moving front of the tubule.

Nematocysts are not only associated with the storage and delivery of jellyfish toxins, but they are also among the most rapid natural delivery systems and serve as the source of jellyfish stings [6]. By investigating the emission mechanism of nematocysts, we can gain insights into the process of stinging, which is beneficial for further prevention and mitigation. Nematocysts stand out as some of the most sophisticated biological micromachines in nature. Understanding their operational mechanism involves analyzing their complex structure and biomechanical transformations. Their ability to function as sophisticated self-assembling biological micromachines makes them an ideal model for biomimetic micro-devices applicable in fields ranging from medical technology to materials science. Upon stimulation, the nematocysts expel stinging needles or stinging cells, resulting in the formation of nematocysts of diverse lengths that serve both predatory and defensive functions. Experimental findings indicate that the emission mechanism of nematocysts is triggered by a synergistic response to mechanical and chemical stimuli, as neither stimulus independently triggers the emission.

In their static state, nematocysts house toxins within a fold, or invagination, on the outer surface of their twisted, folded, and inverted tubules. The lumens of these tubules are filled with internalized barbs. As the nematocysts undergo expulsion and ectropion, toxins are transferred from the outer surface to the inner surface of extended tubules. At the moment of release, toxins accumulate near and inside the base of hollow barbs [7]. In recent years, researchers have conducted extensive investigations into nematocyst emission and achieved significant breakthroughs. Weber et al. [8] first proposed that the osmotic potential of polyglutamate causes the expulsion of nematocysts via osmotic pressure. Nevertheless, previous studies have mainly focused on the initial microseconds of triggering and tubule elongation, neglecting the details of the rest of the process and giving the impression that intracapsular pressure, driven by penetration, provides sufficient force for complete tubule elongation.

To further validate this mechanism, Park et al. [4] introduced an innovative method that employs a mathematical model of mass transfer, combined with a two-channel microfluidic system, to confirm that high osmotic potential is the driving force behind nematocyst tubule movement. Interestingly, the mechanism underlying the stretching process is proposed through a mathematical model of mass transfer. The model assumes that the force required to elongate the tubule is generated by internal pressure and that this pressure increases as the osmotic potential increases.

## 3. Jellyfish Toxins and Biological Activity

The following sections describe the composition and biological activity of jellyfish toxins. Jellyfish stings are closely associated with the composition of their toxins. Upon stimulation, toxins can be injected into the victim through specialized structures known as nematocysts. The venom is composed of a complex mixture, mainly featuring metalloproteinases, phospholipase A2, hyaluronidase, as well as highly intricate proteins and peptide substances. Notably, these include substances such as catecholamines, histamines, and 5-hydroxytryptamine [9], which possess a diverse array of biological effects. They can cause varying degrees of damage to multiple organs. It has been reported that jellyfish toxins can induce organ damage in areas including the blood circulation system, heart, liver, and kidneys. Among the detrimental effects, acute myocardial infarction (heart attack), severe bradycardia (slow heart rate), and hypotension (low blood pressure) are the most critical factors contributing to fatalities from jellyfish poisoning. Researchers believe that the role played by metalloproteinases in jellyfish toxins during the process leading to death should not be underestimated. Unfortunately, the purification of jellyfish toxins is a formidable task due to challenges in identifying novel protein molecules and the lack of a comprehensive understanding of their genome and protein sequences. In this study, we have summarized the primary biological activities of jellyfish toxins and offer theoretical insights that may inform future strategies for prevention and treatment.

### 3.1. Haemolytic Activity

Most of the medusotoxins exhibit hemolytic activity, which has garnered significant attention in the scientific community. The hemolytic proteins within these toxins have been successfully purified and sequenced, representing the only components that have undergone such detailed examination. In 2007, Yu et al. [9] made a groundbreaking discovery that the venom of the jellyfish *Rhopilema esculentum Kishinouye* exhibits significant hemolytic activity, thereby providing novel insights and perspectives into the biological functionality of toxins. Subsequently, Feng et al. [10] identified comparable hemolytic activity in the venom of another jellyfish species, *Cyanea nozakii* (*C. nozakii*), in 2010. These findings gradually led to an understanding that jellyfish venom contains diverse bioactive molecules capable of damaging red blood cells and inducing hemolysis. Li et al. [11] revealed that the hemolytic activity of jellyfish toxins is attributed to a combination of multiple bioactive molecules rather than a single toxic component. Zhang et al. [12] identified the presence of hemolytic proteins in jellyfish toxins and employed bioinformatics methods to classify these proteins as pore-forming toxins that induce cell membrane permeabilization, resulting in the leakage of cellular contents and subsequent hemolysis. This study implies that the mode of action of hemolysin may be associated with the formation of a non-specific cationic channel perforating complex on the erythrocyte membrane [13]. Furthermore, a novel family of CfXT toxin proteins has been identified from various jellyfish toxins, each possessing distinct hemolytic properties [14]. Lazcano-Perez et al. [15] demonstrated the presence of vasoconstrictor components in the extract of *Palythoa caribaeorum* (*P. caribaeorum*) and confirmed its hemolytic and PLA2 activities.

### 3.2. Neurotoxicity

As early as 1990, the neurotoxicity of jellyfish toxin was documented when a patient experienced complete paralysis of the radial nerve, ulnar nerve, and median nerve at the distal end after being stung by a jellyfish [16]. Researchers have gained preliminary insights into the neurotoxicological mechanisms of jellyfish toxins. In the past few years, several jellyfish species such as *Phyllorhiza punctata*, *Cyanea capillata* (*C. capillata*), *Nemopilema nomurai* (*N. nomurai*), and *C. nozakii* have been shown to contain components with neurotoxic properties in their venom [17,18,19]. Burnett et al. [20] have elucidated the profound antagonistic or toxic effects of medulotoxin on the autonomic nervous system, specifically impacting sodium and calcium ion transport, inducing channels or pores in nerve and muscle cell membranes, altering organelle cell linings, releasing inflammatory mediators, binding medulotoxin, and other toxins.

*Chironex fleckeri* (*C. fleckeri*) in the Indo-Pacific region is particularly capable of inducing autonomic nervous disorders in patients by causing peripheral neuropathy and parasympathetic dysfunction. The study conducted by Yang et al. [21] demonstrated, through experimentation on the HT-22 cell line, that venom extracted from *Chrysaora pacifica* (CpV) has the potential to activate Ca^2+^-mediated ROS signaling and induce mitochondrial dysfunction, ultimately leading to neuronal damage or death. Kim et al. [22] discovered that CpV induced persistent peripheral pain, potentially through the activation of Ca^2+^ and ROS signaling cascades in TRPA1 channels. Additionally, CpV significantly augmented primary dorsal root ganglion (DRG) activity. However, the mechanism underlying CPV-induced Ca^2+^ influx into nerve terminal channels remains unresolved. In order to gain further insights into jellyfish toxin activity and composition, Bueno et al. [23] investigated the effects of *Chiropsalmus quadrumanus* extract on sympathetic neurotransmission and found significant interference with norepinephrine neurotransmission. This study contributes to a comprehensive appreciation of the multifaceted nature of jellyfish toxins.

### 3.3. Cardiovascular Toxicity

Cardiovascular toxicity has been a major focus of research on jellyfish venoms. Clinical cases involving jellyfish stings reveal that the vast majority of patients exhibit varying degrees of myocardial damage [24,25]. As early as 1969, studies demonstrated evident cardiotoxicity in animals administered extracts derived from the box jellyfish *Chironex fleckeri* Southcot [26]. In future studies, Freeman et al. [27] chromatographically isolated two unstable high-molecular-weight toxins from the tentacles of *C. fleckeri*, both of which reduced the rate and amplitude of contraction and coronary blood flow in isolated perfused guinea pig hearts. Its cardiovascular effects are thought to be due to direct vasoconstriction, cardiotoxicity, baroreceptor stimulation, and possible vasomotor central inhibition. Subsequently, Mustafa [28] elucidated the potential mechanism underlying the cardiac effects of *C. fleckeri* toxin. Rat cardiomyocytes were subjected to toxin treatment, revealing that the molecular basis for myocardial damage primarily involves Ca^2+^ overload in these cells. Despite these findings, the effects of the diverse components within this venom remain unclear.

Thus, Saggiomo et al. [29] utilized an xCELLigence to quantify cell detachment and discovered that only a particular fraction of *C. fleckeri* venom is cytotoxic to human cardiac muscle cells, with an average molecular weight of approximately 65 kDa. The size range covered by this fraction was found to be around 100–30 kDa. In their pioneering study, Chaousis et al. [30] successfully isolated three distinct cytotoxic components (CTF-α, CTF-β, and CTF-γ) from *C. fleckeri*, which exhibited cardiotoxic effects in vertebrates. These components ranged in size from 13 to 100 kDa. Notably, CTF-α induced rapid but transient toxicity in cells, while CTF-β exerted a slower yet persistent toxic effect. The combined action of these two components displayed a synergistic effect. Furthermore, it was observed that CTF-γ exhibited non-specific toxicity towards both muscle cell types rather than being specific to the heart. Subsequent in vivo studies based on these newly identified toxic constituents have laid the groundwork for additional research and exploration.

Also, the prevalent jellyfish species *C. capillata* found in the coastal waters of southeast China also exhibits certain cardiovascular toxicity; however, its toxic effects are relatively milder than those of *C. fleckeri* and other lethal jellyfish, and it is generally non-fatal. Experimental administration of *C. capillata* tentacle-only extract (TOE) to SD rats resulted in bradycardia, increased T wave amplitude, broadening or even disappearance of QRS waves, followed by a rapid decrease in blood pressure after a transient increase. Blood biochemical analysis revealed a significant elevation in myocardial enzyme levels. Histopathological examination demonstrated severe myocardial injury, corroborated by physical examination findings [31]. However, the precise mechanisms underlying the in vitro direct cardiotoxicity and its toxic action remain elusive. Wang et al. [32] demonstrated a dose-dependent effect of *C. capillata* tenula extract on hemodynamic and electrocardiogram changes using an isolated heart model experiment of Langendorff perfusion, accompanied by a significant increase in heart injury-related enzymes. The overactivation of β- and α-adrenergic receptors in L-type Ca^2+^ channels is postulated to contribute to the development of direct cardiotoxicity. Subsequent experiments further validated the involvement of β-adrenergic receptors in medusotoxin-induced cardiotoxicity and elucidated the underlying mechanism of TOE-induced cardiotoxicity by *C. capillata*. The findings demonstrated that activation of the β-adrenergic receptor/cAMP/PKA signaling pathway contributed to the release of TE from *C. capillata* through intracellular Ca^2+^ overload [33], but further investigation is warranted to elucidate the underlying mechanism. In essence, medusotoxins induce cardiovascular toxicity primarily through intracellular Ca^2+^ overload; however, this process may also involve the formation of non-specific cation channels, excessive activation of L-type Ca^2+^ channels, heightened release of catecholamines, and potential allergic reactions. The extract of *Nemopilema nomurai* (NnV) has also demonstrated cardiotoxicity. Choudhary investigated the impact of NnV on H9c2 cardiomyocytes and confirmed its ability to induce the expression of 25 proteins, including attached protein A2 and myosin 7, as well as upregulate cytokine signaling inhibitor I (SOCS1) and aldose reductase. These alterations can significantly impact the growth, proliferation, and survival of cardiomyocytes, culminating in substantial cellular damage. Notably, there was a notable increase in heat shock protein β1 (HSP27), indicating its potential protective function in safeguarding H9c2 cardiomyocytes against NnV-induced injury [34]. In order to further investigate the pathophysiological mechanism underlying the cardiotoxic effects of jellyfish toxin, researchers utilized sequential chromatography methods to pinpoint the specific cardiotoxic constituents within NnV. Subsequently, a toxic component named NnTP was successfully isolated, and its toxicity was verified through experimentation on zebrafish. The results revealed significant cardiac hemorrhaging. These discoveries provide valuable insights into the precise mechanism by which NnV exerts its cardiotoxic effects, thereby potentially aiding in the development of effective treatment strategies for those suffering from jellyfish stings [35].

### 3.4. Others

In order to further investigate the pathophysiological mechanism behind the cardiotoxicity of jellyfish toxin, researchers employed sequential chromatography techniques to identify the specific cardiotoxic constituents within NnV. Afterward, a toxic component named NnTP was isolated, and its toxicity was verified through experimentation on zebrafish. The results revealed significant cardiac hemorrhaging. These findings offer valuable insights into the precise mechanism by which NnV exerts its cardiotoxic effects, thereby potentially aiding in the development of effective treatment strategies for individuals affected by jellyfish stings. However, under certain circumstances, medusotoxin also has beneficial biological activities, holding considerable pharmacological potential. These properties include anti-inflammatory, antiarrhythmic, antihypertensive, antibacterial, analgesic, as well as anti-cancer and anti-tumor activities. At present, these biological activities are less developed, and with the development of technology, we can turn jellyfish into a valuable resource in the future [36]. These biological activities are well-summarized in Table 1.

In this study, a diverse range of techniques was employed to elucidate the intricate and multifaceted characteristics of jellyfish venom. This research not only deepens our understanding of jellyfish biology but also lays the groundwork for potential therapeutic applications, such as the development of novel antitoxins and biologics. Further studies are expected to shed light on the remaining enigmas of jellyfish toxins, thereby spurring progress in associated scientific and medical domains.

**Table 1:** Summary of Biological Activity. D: Detrimental; B: Beneficial.

Biological Activity	Species	D/B	Refs.
Hemolytic activity	*Rhopilema esculentum Kishinouye*; *Cyanea nozakii*; *Cyanea capillata*; *Palythoa caribaeorum*; *Chironex fleckeri*	D	[9,10,11,12,13,14,15]
Neurotoxicity	*Palythoa caribaeorum*; *Chironex fleckeri*; *Nemopilema nomurai*; *Cyanea capillata*; *Cyanea nozakii*; *Chrysaora pacifica*; *Chiropsalmus quadrumanus*	D	[16,17,18,19,20,21,22]
Cytotoxicity	*Chironex fleckeri*	D	[36]
Cardiovascular toxicity	*Chironex fleckeri*; *Cyanea capillata*; *Nemopilema nomurai*	D	[26,27,28,29,30,31,32,33,34,35]
Muscle toxicity	*Chironex fleckeri*; *Cyanea capillata*	D	[20,37]
Hepatotoxicity	*Cyanea capillata*; *Nemopilema nomurai*	D	[37,38]
Dermal toxicity	*Cassiopea* sp.; *Nemopilema nomurai*	D	[39,40]
Enzyme activity	*Cyanea nozakii*	D/B	[41]
Antioxidant Activity	*Lobonema smithii*; *Chrysaora colorata*	B	[42,43]
Antimicrobial activity	*Catostylus mosaicus*	B	[44]

## 4. Symptoms of Jellyfish Stings

### 4.1. Local Symptom

**Figure 2:**
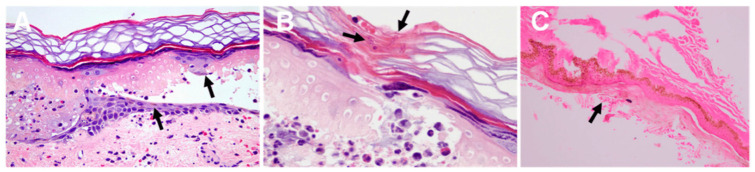
The skin is pathological after jellyfish stings [49]. (**A**) epidermal necrosis and vesicle formation following a *Pelagia noctiluca* sting. Pigmented keratinocytes (black arrows), vasodilatation, edema, and erythrocyte extravasation are displayed; (**B**) fragments of nematocyst tubules are shown in the stratum corneum (black arrow); (**C**) remains of nematocysts (arrow) following a *C. fleckeri* sting.

Jellyfish stings are largely characterized by skin-related symptoms. Upon contact with the tentacles of a jellyfish, individuals typically experience immediate local reactions such as burning and stinging pain, which are often paired with a discoloration of the skin at the point of contact, ranging from reddish to brownish or purplish hues. Less severe stings manifest as focused erythema, small papules, and linear welts that mirror the direction of the tentacle’s touch, creating lesions that appear like whip lashes. These symptoms are commonly accompanied by significant itching. In severe cases, subsequent stages may present as edematous masses, urticaria, blister formation, and erosion, along with hemorrhage and necrosis within the affected tissue [45]. Histopathological examination of the lesions may reveal both nematocyst fragments and characteristic inflammatory infiltrates (Figure 2A–C).The clinical manifestations of jellyfish stings may resolve within a few weeks, while the associated pain and treatment can persist for weeks or even years. In certain cases, patients may develop type IV allergic hypersensitivity, characterized by delayed or recurrent skin lesions either at the primary site or distant locations [46].

Jellyfish stings frequently affect the eyes and face. Ocular jellyfish sting is an infrequent ophthalmic emergency encountered in clinical practice. The predominant clinical manifestations include pain, conjunctival congestion, keratopathy, and photophobia. Furthermore, the protracted course of the disease may give rise to complications such as iritis, elevated intraocular pressure, mydriasis, impaired accommodation, and the formation of peripheral adhesions [47]. The severe complications that can arise from jellyfish stings include optic nerve atrophy, occlusion of retinal blood vessels, retinal thinning, and scarring in the macular region [48].

### 4.2. Systemic Symptom

The composition of medusotoxin is highly intricate, involving numerous cells and organs, thereby easily inducing symptoms of systemic poisoning. Systemic reactions can occur within minutes to hours following moderate and severe stings, primarily encompassing vertigo, dyskinesia, spastic or flashover paralysis, multiple neuritis, syncope; hemolysis, arrhythmia, hypotension, congestive heart failure; diffuse muscle joint pain or spasticity, tonia rectus abdominis; nausea, vomiting, and diarrhea; as well as allergic pulmonary edema [50] anaphylactic shock, acute pulmonary heart disease and kidney failure, until death. Buscetta et al. documented the first case of severe rhabdomyolysis induced by jellyfish stings in the Mediterranean Sea, underscoring the significance of prompt identification and management of jellyfish-induced rhabdomyolysis [51]. The Irukandji syndrome is a painful and potentially fatal condition induced by jellyfish, such as *Carukia barnesi*, which can be found in up to 25 species. The initial sting may go unnoticed due to its mild nature. However, severe systemic symptoms typically emerge within the first 30 min after being stung, with a range of onset varying from 5 to 120 min. The initial signs of Irukandji syndrome include systemic symptoms like back pain, diffuse muscle cramps, nausea, vomiting, excessive sweating, headache, anxiety, agitation, and piloerection. These are followed by frequent hypertension and tachycardia. In severe instances, heart failure in conjunction with pulmonary edema can result in respiratory failure and potentially lead to death [52].

## 5. Treatment of Jellyfish Stings

### 5.1. Diagnosis

**Figure 3:**
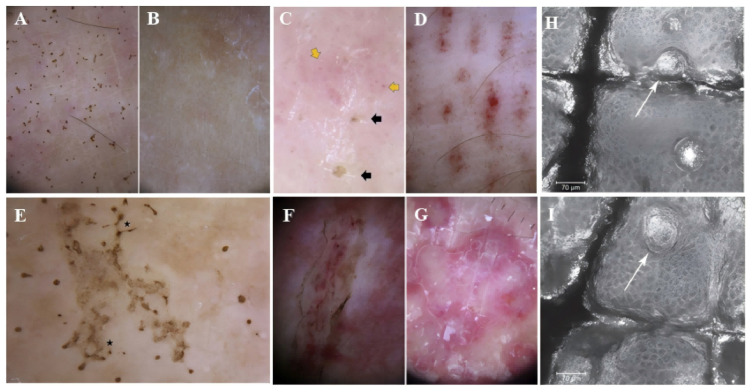
Dermoscopy of Pelagia noctiluca jellyfish stings: (**A**) Tingling for 1 week, 0.1 mm brown spot; (**B**) Brown spot disappeared at 21 days in same lesion; (**C**) Brown crusts (black arrows) & red dots (yellow arrows) on pink; (**D**) “Linear purpura”: linear band of regularly spaced red dots in tabby pattern; (**E**) “Chinese character” pattern: brown dots joined by light brown granular lines (★), matching linear vesicular disease variant; (**F**) “Snake ulcer”. Scales & brown dots mark margin, with internal linear purpura; (**G**) “Round opalescent red area” due to repeated, persistent inflammatory response to sting; [53] (**H**) RCM shows multiple special structures (arrow) through epidermis, corresponding to cnidocysts; (**I**) Another RCM slice (frontal epidermis view) displays harpoon structure (cnidocyst) passing through epidermis (arrow) [55].

The diagnosis of jellyfish stings is primarily clinical in nature. When a clear history of jellyfish contact is not present, the resulting skin lesions may be mistaken for conditions such as herpes, impetigo, phytodermatitis, other Marine animal stings, or other whipping skin diseases, potentially leading to delays in both diagnosis and treatment. Dermoscopy and Reflectance Confocal Microscopy (RCM) are non-invasive techniques employed for diagnosing jellyfish stings. In a study examining dermoscopy of Pelagia noctiluca stings, four characteristic dermoscopic features were identified: brown dots, brown “hanji” patterns, needle-like brown projections, and white-yellow crusts. In cases where there is no reported jellyfish encounter, a dermoscope can serve as a valuable diagnostic tool for jellyfish stings [53] (Figure 3A–F). Di Stefani et al. outline the application of dermoscopy and RCM in determining the clinical diagnosis and managing patients with jellyfish stings. Dermoscopic evaluation revealed multiple brown spots and lines, well-defined brown erosions, and localized scales distributed on a pink background similar to those previously mentioned. In addition, RCM imaging distinctly revealed the presence of highly coiled hollow, harpoon-like structures, consistent with nematocysts, inserted into the upper layer of the epidermis [54]. Compared with the results of Di Stefani et al., the RCM analysis conducted by Paolino et al. identified diffuse partial super reflection along with hollow and harpoon-like structures [55] (Figure 3H,I). These distinctive characteristics assist in facilitating an accurate diagnosis.

### 5.2. Local Therapy for Mild Conditions

#### 5.2.1. Stay Away from Stings

Immediate disengagement from the jellyfish is crucial after a sting. Following stings, remnants of jellyfish tentacles and venom persist in the skin, and attached tentacles can continue to inject venom via their nematocysts. Therefore, prior to treatment, tweezers can be employed for extracting the tentacles, or alternatively, gentle removal using gloves or other protective measures should be undertaken to avoid squeezing and eliminate residual tentacles from the skin.

#### 5.2.2. Local Application

Lidocaine is a commonly employed local anesthetic that is effective in reducing or eliminating pain in specific areas. In some cases, lidocaine may be used therapeutically to relieve discomfort and symptoms caused by jellyfish stings. A systematic review by Ward and colleagues on venom-induced injuries caused by jellyfish and related organisms in North America and Hawaii has recommended the application of diluted solutions of the local anesthetic lidocaine as an efficacious approach for ameliorating pain associated with jellyfish stings. The use of lidocaine at concentrations of 10% and 15% has been demonstrated to rapidly relieve tingling sensations [56,57]. Ballesteros et al. [58] introduced a novel potential inhibitor, comprised of 10% lidocaine in ethanol solution, 1.5% hydroxyacetophenone, and butanediol in distilled water plus butanediol, which is conducive to inhibiting the emission of jellyfish nematocysts, offering a fresh perspective. Conversely, some studies indicate that local anesthetics, such as lidocaine, exhibit limited efficacy in the absence of obstructive bandages and may demonstrate delayed or even toxic effects when applied to extensive areas [59]. Lidocaine is not considered a standard therapy for jellyfish stings and should only be used with the supervision and recommendation of a healthcare professional. Moreover, over-the-counter analgesics such as acetaminophen or ibuprofen can effectively alleviate pain from jellyfish stings. These medications possess the ability to decrease both pain and inflammation, aiding patients in managing the discomfort associated with stings. However, it is crucial to note that analgesics should not take the place of appropriate first-aid procedures and should be used in conjunction with other treatments.

#### 5.2.3. Hot/Cold Water

Hot water is widely regarded as one of the most efficacious treatments for non-tropical jellyfish stings [60]. The use of hot water can result in clinically significant pain reduction. Immersion in hot water at approximately 45 °C is a well-documented and generally recommended therapeutic approach. A recent systematic study has offered substantial evidence to endorse hot water immersion to alleviate pain, emphasizing that soaking the affected area in 45 °C water for 20 min represents the most effective treatment option [61]. Nevertheless, despite its preferred status, the precise mechanism underlying this modality remains unclear, necessitating further investigation [62]. While hot water immersion is typically favored, cold water may be more appropriate for certain tropical jellyfish stings. Cold water effectively decreases local swelling and alleviates pain associated with puncture wounds from tropical species such as box jellyfish [60]. Applying ice packs, ice cubes, or immersing the affected area in cold water can effectively reduce inflammation and slow the spread of toxins [63]. It is important to note, however, that cold water therapy is not universally applicable to all jellyfish stings; caution must be exercised due to potential warming effects on some jellyfish toxins, which could exacerbate toxic reactions.

#### 5.2.4. Acetic Acid (Vinegar) Treatment

Acetic acid (vinegar) rinses have been used in Chinese folklore to treat jellyfish stings, involving the cleansing of the affected area following envenomation.

However, the efficacy of vinegar treatment for jellyfish stings remains a topic of debate. Doyle et al. observed that rinsing *C. capillata*’s tentacles with vinegar unexpectedly resulted in venom release from the nematocysts but reduced venom activity [64]. Additionally, other studies have demonstrated that acetic acid inhibits discharge activation, and acidic pH helps mitigate the harm caused by jellyfish stings [65]. The application of acetic acid as a therapeutic measure for certain jellyfish stings, such as those caused by lion’s mane jellyfish, is still debated due to its potential to neutralize toxins released by some species while potentially exacerbating symptoms in others, particularly box jellyfish. Therefore, when considering adjunct therapy using acetic acid, it is essential to conduct a meticulous evaluation of the particular jellyfish species involved.

When dealing with jellyfish stings, it is crucial to dispel some common misconceptions. For instance, rinsing the wound with fresh water may actually intensify the pain by provoking a greater release of jellyfish venom. Furthermore, studies on box jellyfish have demonstrated the ineffectiveness or even harmful effects of urea on post-sting wounds, as it can significantly enhance nematocyst endotoxin excretion [66]. It is also important to avoid direct contact with the affected area to prevent exposure to unreleased toxins. In summary, the supportive treatments for jellyfish stings encompass detaching from the sting source, local application of medicine, hot/cold water, and acetic acid (vinegar) treatment. However, there remains a significant knowledge gap in the supportive care for jellyfish stings. Current research remains controversial, and further systematic studies are needed to clarify the efficacy and appropriateness of various treatment modalities. Gaining a comprehensive understanding of the role of supportive treatments in managing jellyfish stings can not only assist patients in alleviating the discomfort associated with stings but also guide future investigative efforts.

### 5.3. Systemic Treatment (Severe)

#### 5.3.1. Antivenom Therapy

Antivenom may stand out as a promising treatment approach for severe jellyfish stings. In 1970, the Australian Commonwealth Serum Laboratory (CSL) successfully isolated gamma globulin (IgG) from plasma obtained from *C. fleckeri* toxin-immunized sheep, leading to the development of CSL box jellyfish antivenom [67]. This antivenom is the only commercially available treatment for jellyfish toxins to date. However, its efficacy against cardiovascular toxicity is limited and requires prior administration in combination with jellyfish toxins [68,69]. Andreosso A et al. [70] conducted a comprehensive analysis of the dose and time dependence of CSL box jellyfish antivenoms using cell assays, providing valuable insights for their application. Piontek et al. [71] elucidated the mechanism of action underlying *C. fleckeri* venom, thereby contributing to the development of novel therapeutic strategies. Furthermore, experimental evidence has demonstrated that the combination therapy involving magnesium sulfate and *C. fleckeri* antitoxic serum effectively prevents cardiovascular failure [72]. Geographical variations in animal toxins are prevalent, and the efficacy of antiserum exhibits regional disparities. Tailoring region-specific antivirals is imperative for addressing these differences, the complex structure and firing mechanism offer valuable insights in the fields of cell biology and toxicology, laying a theoretical foundation for preventing jellyfish stings from the source Yu et al. [73] comprehensively summarized the venom constituents and primary biological activities of distinct venoms from the same species, offering a valuable scholarly reference. *C. nozakii* jellyfish, found off the coast of China, are responsible for causing numerous stings and even fatalities annually. Regrettably, there is currently a lack of effective treatment options for severe stings. Consequently, the development of an antivenom specific to this jellyfish species has become an urgent priority. New Zealand white rabbits were immunized with *C. nozakii* toxin to obtain antitoxic serum, from which IgG (AntiCnTXs) was isolated using protein A resin. The purified and optimized F(ab’)2 antitoxic serum [F(ab’)2-anticntxs] was obtained by pepsin digestion of AntiCnTXs. It exhibited a significant neutralizing effect on the toxins. This research not only provides valuable insights into the preparation of antivenom against medusotoxin but also offers potential directions for future production of therapeutic drug [74,75] With the advancement of science and technology, there has been a profound deepening in the exploration of jellyfish toxins, facilitating an enhanced comprehension and utilization of these toxins while ensuring improved safeguarding of marine ecosystems and human well-being.

#### 5.3.2. Metalloproteinase Inhibitor

**Figure 4:**
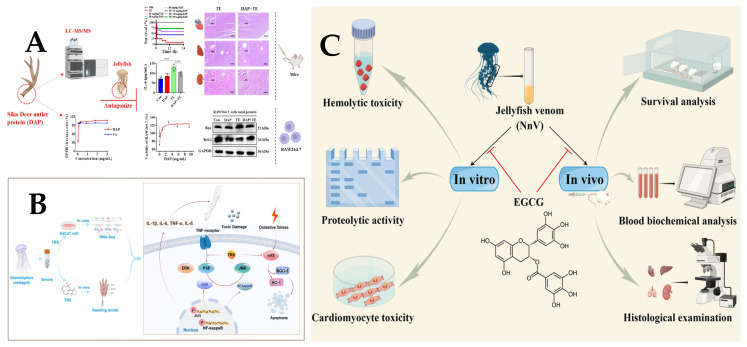
(**A**). Sika Deer antler protein antagonizes the inflammatory response and oxidative damage induced by jellyfish venom [99]. **** *p* < 0.001. (**B**). Troxerutin suppresses the inflammation response and oxidative stress in jellyfish dermatitis by activating the Nrf2/HO-1 signaling pathway [100]. (**C**). Protective Effects of Epigallocatechin-3-gallate (EGCG) against the Jellyfish *Nemopilema nomurai* Envenoming [91].

Jellyfish toxins contain a significant abundance of metalloproteinases (MMPs), a class of proteases that depend on metal ions for catalytic activity at their active sites. Excessive MMP levels in pathological conditions can lead to tissue remodeling disorders and contribute to the development of various diseases. A recent study by Li et al. [76] utilized transcriptomic and proteomic analyses to examine Stomolophus meleagris-related toxins, identifying the presence of 33 dominant metalloproteinases that play a crucial role in S. meleagris stings. Furthermore, the presence of metalloproteinases in various jellyfish venoms [17,77,78,79,80] has been unequivocally confirmed, establishing them as the principal venom components. It is believed that metalloproteinases play a key role in inducing jellyfish toxicity. Consistent with this, Lee et al. [81] demonstrated a positive correlation between *N. nomurai* activity and its cytotoxicity through cellular experiments. Yu et al. [82] conducted a comprehensive analysis using skin proteomics and serum metabolomics to investigate the impact of toxic metalloproteinases on jellyfish sting-induced dermatitis. Their findings underscored the significant role these enzymes play in triggering the condition, offering new insights for future research. A recent study has identified 29 and 25 metalloproteinases at the transcriptome and proteome levels, leading to the isolation of two zinc metalloproteinases, namely DN6695_c0_g3 and DN8184_c0_g7. This finding further supports the notion that metalloproteinases play a pivotal role as major toxins in jellyfish [83]. Metalloproteinases are also known to contribute to proinflammatory activity, edema formation, myotoxicity, lethality, and venom-related pathogenesis [84,85,86,87]. Consequently, targeting metalloproteinase inhibition could serve as a promising therapeutic strategy for mitigating medusotoxin toxicity. Batimastat (BMT) is an iso-hydroxamic acid peptide mimetic that selectively binds to zinc ions within metalloproteinases, thereby specifically inhibiting their enzymatic activity. Therefore, Li et al. [88] investigated the therapeutic potential of BMT and EDTA in the treatment of jellyfish stings, demonstrating their ability to attenuate cellular expression levels of inflammatory factors IL-6, TNF-α, and MCP-1. Experimental findings have demonstrated that the administration of the metalloproteinase inhibitors BMT and EDTA can significantly mitigate the inflammatory stimulation and necrosis of muscle tissue induced by NnV venom [87]. Wang et al. [89] have suggested that BMT (BB-94) could effectively inhibit metalloproteinase-induced vascular injury and ameliorate liver and kidney hemorrhagic injury, thereby alleviating jellyfish stings. In addition to synthetic metalloproteinase inhibitors, natural polyphenols also hold potential value in the therapeutic management of jellyfish stings due to their wide distribution in the plant kingdom and ability to bind and precipitate proteins, leading to inhibition of proteolytic activity with diverse applications. Epigallocatechin-3-gallate (EGCG), the primary constituent of green tea polyphenols, has been demonstrated by Hwuang et al. [90] in both in vivo and in vitro models to attenuate or eliminate NNV-induced MMP-2 and MMP-9 activity and expression. Additionally, EGCG reduces neutrophil accumulation and bleeding while promoting regeneration at the site of injury, suggesting its potential as a therapeutic agent for NNV-induced skin inflammation [91] (Figure 4C). With the advancement of technology, Asirvatham et al. [92] employed pharmacoinformatics to meticulously select 39 distinct flavonoids and successfully identified silymarin as a potent inhibitor of NnV-MP through comprehensive computer-assisted 3D structure prediction, molecular docking, and molecular dynamics analysis.

The robust inhibitory effect of silymarin can be primarily due to its remarkable hydrophobic affinity and optimal hydrogen bonding capabilities. Furthermore, the significant antioxidant and anti-inflammatory biological activities of phenolic acids (hydroxybenzoic acids) have garnered considerable attention from researchers. Geng et al. [93] identified protocatechuic acid (PCA) and gentianic acid (DHB) as potential therapeutic agents for jellyfish stings to mitigate NnV-induced damage. Their research revealed that both compounds effectively neutralized metalloproteinases and reduced their toxicity, highlighting the promising application of phenolic acids in the treatment of jellyfish stings. The exploration of whether the secondary metabolites of Marine symbiotic fungi can inhibit jellyfish venom enzyme activity remains incomplete, although the development of inhibitors from the ocean has emerged as a promising approach. Symbiotic fungi in marine invertebrates such as sponges, anemones, and corals have demonstrated the ability to produce diverse secondary metabolites with distinctive structures and unprecedented biological activities. However, it is necessary to address certain limitations in previous studies. For instance, Yang et al. [94] identified phenazine-1-carboxylic acid (PCA) from the meduse-associated fungus Aspergillus SmT07 as a novel inhibitor of NnV metalloproteinases; however, their study did not employ purified NnV metalloproteinases for in vitro screening assays nor verify the in vivo inhibition of PCA on jellyfish venom-induced toxicity. Future efforts should focus on addressing these issues to precisely identify more specific and effective inhibitors of NnV metalloproteinases that could contribute to the development of therapeutics against jellyfish venom.

#### 5.3.3. Anti-Allergy Medications or Antibiotics

Allergic reactions caused by jellyfish stings, which can manifest as urticaria, necessitate the administration of anti-allergic medications including cyproheptadine, loratadine, and midazolastine [95]. These drugs effectively alleviate symptoms associated with allergic reactions such as itching, urticaria, and swelling. In cases of severe allergic reactions, oral or topical corticosteroids like hydrocortisone or anti-sensitivity creams can be employed. A small subset of patients experiencing jellyfish stings may encounter life-threatening anaphylactic shock; in such instances, immediate utilization of epinephrine is crucial to stabilize blood pressure and respiration. If a jellyfish sting leads to infection development, antibiotic treatment may be necessary [96].

#### 5.3.4. Chinese Medicinal Therapy

Jellyfish stings belong to the venomous category of Chinese medicinal insects and animals. In recent years, the clinical use of traditional Chinese and Western medicine to treat jellyfish stings has been explored. Declerck et al. [97] demonstrated the effective pain relief provided by papain for patients suffering from jellyfish stings. Barth et al. [98] extracted a hydroethanolic extract from the plant *Ipomoea pes-caprae,* which alleviated mouse discomfort caused by jellyfish stings and exhibited anti-inflammatory properties. The key to combining Chinese and Western medicine for treating jellyfish stings lies in addressing both symptoms and underlying causes while integrating internal and external approaches. Zhang et al. [99] showed that Dier antler protein (DAP) can effectively antagonize the toxicity of jellyfish toxins, highlighting the potential of Chinese medicine in the treatment of jellyfish stings (Figure 4A).

#### 5.3.5. Others

The inhibition of perforin inhibitors (PFPs) and Ca^2+^ antagonists could potentially serve as therapeutic modalities. PFPs, being the primary active molecules in jellyfish toxins, induce hemolysis and cytotoxicity upon jellyfish stings. Consequently, suppressing the activity of PFPs can mitigate the hemolytic and cytotoxic effects of jellyfish toxins, thereby alleviating symptoms associated with jellyfish stings. Feng et al. [10] studied cell membrane surface components, carbohydrate, lecithin, cholesterol, and sphingomyelin pairs. The study revealed that sphingomyelin could render *C. nozakii* hemolytic toxicity by competitive binding, resulting in a 70% reduction. Additionally, Ca^2+^ channels may be implicated in cardiac toxicity; henceforth, Ca^2+^ antagonists hold promise as future therapeutic agents for treating jellyfish stings. Kim et al. [22], through their research on *Chrysaora pacifica* jellyfish toxins-induced peripheral pain linked to inflammation and neurotoxic oxidative signaling pathways, demonstrated that verapamil, TTA-P2, and T-type Ca^2+^ channel blockers effectively relieved pain caused by these toxins. Moreover, significant pain reduction was also observed with the use of Ca^2+^ chelating agents (EGTA and BaptaAM), providing a foundation for employing Ca^2+^ channel blockers in mitigating damage induced by jellyfish toxins.

Inflammation and oxidative stress are recognized as pivotal pathogenic mediators in jellyfish envenomation. Troxerutin (TRX), a hydroxymethylated flavonoid derivative, demonstrates multifaceted bioactivities, including potent suppression of both inflammatory cascades and oxidative damage pathways [100] (Figure 4B).Furthermore, the research by Yanagihara et al. [101] indicates that cardiovascular failure induced by Cubozoan venom is caused by hyperkalemia and can be prevented by zinc gluconate in mice. Subsequently, potassium channel toxins may be present among the key lethal toxins isolated from *C. nozakii* [41]. Therefore, the regulation of potassium should be an important issue that needs to be urgently addressed after a jellyfish sting. The information related to treatment strategies is shown in Table 2.

## 6. Conclusions and Future Prospects

Jellyfish stings are a common marine biological injury, yet the complexity of jellyfish toxins renders them difficult to purify and isolate, and there is still a lack of targeted drugs and suitable treatment plans. Jellyfish stings are still a major problem that needs to be solved by many scientific researchers. This review originates from the structure of the emission mechanism, the most prominent type of nematocyst in jellyfish, to explore the firing mechanism of jellyfish nematocysts. Such an investigation contributes to a better understanding of the unique biological structures and functions of jellyfish. Its complex structure provides a reference for targeted drug delivery and biomimetic microneedles, and lays a theoretical foundation for preventing jellyfish stings at the source. In addition, this review also analyzes the biological activities of jellyfish toxins. On one hand, clarifying the biological activities of jellyfish toxins is conducive to the development of more targeted antivenom drugs and effective treatment methods. On the other hand, through a comprehensive understanding of the biological activities, the potential values of jellyfish toxins in biological research and development can be explored. The understanding of jellyfish sting symptoms serves as the foundation for medical diagnosis and treatment. Symptoms such as local pain, swelling, and, in some cases, systemic symptoms like dizziness, weakness, and breathing difficulties. Medical staff can quickly diagnose whether a patient is stung by a jellyfish and the degree of the sting based on these specific symptoms, and then adopt corresponding first-aid and follow-up treatment measures. More notably, the sting symptoms provide a reference value for scientific researchers to further study the impact of jellyfish toxins on human physiological functions. The research results can also complement and draw on other studies related to injuries and diseases caused by marine organisms, promoting the continuous development of this field. Furthermore, this article also summarizes the current treatment strategies for jellyfish stings, which have laid a solid foundation for the treatment of jellyfish stings. However, there are still very few specific drugs for jellyfish stings at present. Except for specific antidotes for some highly toxic jellyfish, symptomatic treatment drugs are mainly used for most jellyfish stings. Although these drugs can relieve symptoms, they cannot completely eliminate the effects of toxins. Especially for some unknown toxins or stings from new types of jellyfish, the therapeutic effects of drugs may not be satisfactory.

Nevertheless, the current treatment methods still have significant limitations. In response to these deficiencies, on one hand, we need to strengthen scientific education and enhance public awareness. Through education, people can understand the biological characteristics, living habits, and toxicity of different jellyfish species. A profound understanding of jellyfish biology enables better identification of potential risks and the adoption of preventive measures to reduce the occurrence of stings. On the other hand, we should continue to strive based on previous research, develop more accurate and rapid diagnostic tools, and intensify the research and development of specific antidotes for a broader range of jellyfish species. We believe that with the advancement of science and technology, the main active components of jellyfish toxins will be continuously identified. The drugs developed based on the mechanisms of these toxins will play a crucial role in the treatment of jellyfish stings in the future.

## Figures and Tables

**Table 2:** Summarize treatment strategies.

Systemic Treatment (Severe)	Approach	Refs.
Antivenom	*C. fleckeri* antitoxic serum	[67]
	F(ab’)2-AntiCnTXs	[74,75]
Metalloproteinase inhibitor	Batimastat (BMT)	[88]
	EDTA	[87]
	EGCG	[90]
	Silymarin	[92]
	PCA and DHB	[93]
	PCA	[94]
Anti-allergic medicine	Urticaria, Necessitate the administration	[95]
antibiotics	Hydrocortisone	[96]
Chinese medicinal therapy	Papain	[97]
	Hydroethanolic extrac	[98]
	DAP	[99]
PFPs inhibitor	Sphingomyelin	[10]
Ca^2+^ antagonists	Verapamil; TTA-P2; T-type Ca^2+^ channel blockers	[22]
K^+^ antagonists	Zinc gluconate	[101]

## Data Availability

Data are contained within the article.
